# Editorial

**Published:** 1853-08

**Authors:** 


### THE MEDICAL EXAMINER.

PHILADELPHIA, AUGUST, 1853.

### DEATH OF NATHANIEL CHAPMAN, M. D.

The declining health of Professor Chapman, for some years past, and his advanced age, had long since prepared us for the announcement of his death, which took place in this city on the 1st of July last. He had been, for the last three years, entirely withdrawn both from professional duties and general society, and his death was the result rather of a slow and gradual decay than of any positive disease.

Dr. Chapman was a native of Virginia, where he was born, in Fairfax Co., on the 28th of May, 1780. His family was of an old and respectable English stock; his paternal ancestor, who came to Virginia with the first colony, having been a captain of cavalry in the British army, and the youngest son of a cousin-german of Sir Walter Raleigh. The family settled on the river Pomonkey, some twenty miles from Richmond; but the branch from which the Doctor is descended, migrated, about a century and a half ago, to Maryland, and fixed itself on an estate on the banks of the Potomac, nearly opposite Mount Vernon, which is still, we believe, in their possession. The Doctor's father, however, went to Virginia upon his marriage, where he afterwards remained.

Dr. Chapman received his early education at the Classical Academy of Alexandria, founded by General Washington, where he passed six years. He subsequently spent a short time in two colleges, though not long enough, as we have heard him say, to owe either any obligation. He came to Philadelphia in the autumn of 1797, and commenced the study of medicine with the late Professor Rush, of whom he became a a favorite pupil. He continued three years in the office of Dr. Rush, and in attendance upon lectures at the University of Pennsylvania, from which he received his degree in the spring of 1800. The Doctor's thesis was upon *hydrophobia*, written at the request of Dr. Rush, in answer to an attack upon his favorite theory of the pathology of that disease. Dr. Chapman had previously prepared another thesis, on the "Sympathetic connections of the stomach with the rest of the body," which he afterwards read before the Philadelphia Medical Society. This contained the substance of his peculiar views on fever and other diseases, as well as the modus operandi of medicines. While a student, Chapman found time to become a contributor to the Portfolio, a magazine of some celebrity in its day. His contributions, under the signature of Falkland, obtained considerable popularity.

In 1801, Dr. Chapman went abroad, where he spent four years—chiefly in Edinburgh and London. He remained a year in London, the private pupil of Abernethy, and thence passed to Edinburgh. Here he not only turned to account the advantages of the then celebrated school of medicine, but he enjoyed also the privilege of free intercourse with the distinguished literary and scientific circles of the modern Athens. He became intimate with many of the eminent persons of those days, among whom may be mentioned Dugald Stewart, the Earl of Buchan, and Mr. (afterwards Lord Chancellor) Brougham,* then a fellow student. Before Dr. Chapman's departure from Edinburgh, Lord Buchan gave him a public breakfast, on the birthday of Washington, at which a number of distinguished persons were present, when he took the occasion to entrust him with an interesting relic, valuable from a double historical association. Lord Buchan had presented to General Washington a box made of the oak that sheltered Sir William Wallace, after the battle of Falkirk, with a request to "pass it, in the event of his decease, to the man in his country who should appear to merit it best." General Washington, declining so invidious a designation, returned it by will to the Earl, who committed it to Dr. Chapman, to be delivered to Dr. Rush, with a view

* In 1809, Dr. Chapman republished, in Philadelphia, Mr. Brougham's speech in the House of Commons, on the British Orders in Council, with a biographical sketch of him, in which he predicted his future eminence. Lord Brougham was then quite a young man, little known in this country.

to its being ultimately placed in the cabinet of the College at Washington, to which General Washington had bequeathed a large sum.

Dr. Chapman returned to the United States in 1804. He established himself in Philadelphia, and in 1808, formed a matrimonial connection with Rebecca Biddle, daughter of Col. Clement Biddle, from which, during a period of nearly fifty years, he derived the highest degree of domestic happiness. His success in practice was rapid, and he became almost immediately the favorite physician of a large portion of the higher classes of Philadelphia, with whom he enjoyed a popularity as a practitioner, which has, perhaps, never been equalled here. A rare combination of personal qualities—attractive manners, wit, great conversational powers, and the kindest feelings—made Dr. Chapman as personally popular as he was professionally eminent.

As a writer and lecturer, Dr. Chapman long occupied a high rank. Soon after his return home, he published a work entitled "*Select Speeches, Forensic and Parliamentary,*" with critical and illustrative remarks, in five 8vo. volumes, which attracted much attention. He afterwards, however, confined his pen to scientific topics.

The year of his return to the United States, 1804, he gave a private course of lectures on Obstetrics, which proved so popular that, in 1806, at the age of twenty-six, he was elected adjunct to the Professor of Midwifery in the University of Pennsylvania, and soon afterwards he was elected to fill the chair of the Materia Medica. At the death of Rush, in 1812, he was transferred to the chair of Theory and Practice, which he filled from that date till his resignation in 1850. Professor Chapman's lectures, delivered as they were annually to large classes, for a period of nearly forty years, are of course familiar to no small portion of the profession of the United States; and it is almost superfluous in us to speak of them as erudite, elaborate and highly finished compositions, enriched with the stores of the most varied reading and the largest personal experience. They have been given to the public in various forms; many of them were published at length in the early volumes of this journal, and a compendium of them was subsequently issued, under the editorial supervision of Dr. N. D. Benedict, now of New York. Dr. Chapman's delivery of his lectures was animated, graceful, and emphatic. His voice had a slight nasal intonation, which was not unpleasant to the ear when familiarized with it. His lectures were read from the manuscript, but were frequently pointed and enlivened by the extemporaneous introduction of apt anecdote and humorous illustration.

In addition to his course at the University, Dr. Chapman for a long period gave clinical lectures in the hospital of the Philadelphia Alms House. For nearly twenty-five years, also, he delivered a summer course of lectures in the Medical Institute of Philadelphia. This institution, which is still in existence, and is the oldest in the United States of the kind, was founded by Dr. Chapman, although he generously declined all participation in the fees, or control over the appointments to chairs.

Dr. Chapman has filled numerous honorable appointments in medical and learned societies. He frequently occupied the post of President of the Philadelphia Medical Society, and was the successor to Duponceau in the eminent distinction of the Presidency of the American Philosophical Society.

Dr. Chapman's principal work is his "*Therapeutics*," published in 1817, which went through seven editions, one of them surreptitious. This treatise long maintained distinguished popularity among the works on Materia Medica, and now occupies a high rank as a book of reference. The author's views, however, on the modus operandi of medicines are of the ultra-solidist scoool, and, it is hardly necessary to add, not in accordance with received opinions.

In 1820 Dr. Chapman commenced the publication of the "Philadelphia Journal of the Medical and Physical Sciences," which he continued to edit for many years. The journal was undertaken with liberal views—the Doctor never receiving any salary for his services. He was subsequently an occasional contributor to different periodicals.

In the foregoing sketch we have presented a brief notice of the leading events in the professional career of Dr. Chapman. We leave, however, to other hands to do justice to the eminent social qualities which so long endeared him to all classes of the city in which the greater portion of his life was spent.

---

At the semi-annual meeting of the Faculty of Jefferson Medical College, held on the second day of July, 1853, the announcement of the decease of Professors HORNER and CHAPMAN having been made, it was resolved *unanimously*, that the Faculty, in common with their medical brethren, deeply deplore the loss to science of two individuals, the better part of whose valuable lives had been spent in the successful teaching of a profession of which they were distinguished ornaments, and to the advancement of which they had both so largely contributed.

Resolved, That a copy of this resolution be sent to the families of the deceased, and be published in the medical journals.

Extracted from the minutes. R. M. HUSTON, M. D.,
*Dean of the Faculty.*

